# The Biosynthesis of Unusual Floral Volatiles and Blends Involved in Orchid Pollination by Deception: Current Progress and Future Prospects

**DOI:** 10.3389/fpls.2017.01955

**Published:** 2017-11-13

**Authors:** Darren C. J. Wong, Eran Pichersky, Rod Peakall

**Affiliations:** ^1^Ecology and Evolution, Research School of Biology, The Australian National University, Canberra, ACT, Australia; ^2^Department of Molecular, Cellular and Developmental Biology, University of Michigan, Ann Arbor, MI, United States

**Keywords:** *Orchidaceae*, pollination, floral volatile, biosynthesis, semiochemical, deception, volatile organic compounds (VOCs), evolution

## Abstract

Flowers have evolved diverse strategies to attract animal pollinators, with visual and olfactory floral cues often crucial for pollinator attraction. While most plants provide reward (e.g., nectar, pollen) in return for the service of pollination, 1000s of plant species, particularly in the orchid family, offer no apparent reward. Instead, they exploit their often specific pollinators (one or few) by mimicking signals of female insects, food source, and oviposition sites, among others. A full understanding of how these deceptive pollination strategies evolve and persist remains an open question. Nonetheless, there is growing evidence that unique blends that often contain unusual compounds in floral volatile constituents are often employed to secure pollination by deception. Thus, the ability of plants to rapidly evolve new pathways for synthesizing floral volatiles may hold the key to the widespread evolution of deceptive pollination. Yet, until now the biosynthesis of these volatile compounds has been largely neglected. While elucidating the biosynthesis in non-model systems is challenging, nonetheless, these cases may also offer untapped potential for biosynthetic breakthroughs given that some of the compounds can be exclusive or dominant components of the floral scent and production is often tissue-specific. In this perspective article, we first highlight the chemical diversity underpinning some of the more widespread deceptive orchid pollination strategies. Next, we explore the potential metabolic pathways and biosynthetic steps that might be involved. Finally, we offer recommendations to accelerate the discovery of the biochemical pathways in these challenging but intriguing systems.

## Introduction to Deceptive Pollination

Flowers have evolved a diverse array of strategies to secure pollination, with both visual and olfactory cues (i.e., pigmentation and scents) often proving crucial long-distance signals to potential pollinators (Pichersky and Gershenzon, 2002; Raguso, 2008; Muhlemann et al., 2014). Lured by visual and olfactory cues, animal visitors to flowers can be broadly categorized into three groups: non-pollinator visitors that exploit plant reward – nectar, pollen, or other tissue – but do not pollinate the flower, pollinator visitors that secure reward for their service of pollination; and pollinator visitors that are deceptively exploited by the plant without reward (Schiestl and Schlüter, 2009).

Although by no means exclusive to orchids, deceptive pollination strategies are particularly well-developed in the *Orchidaceae* with an estimated one third of the family (∼10,000 species) using such strategies (Jersáková, 2006). For example, the flowers of some deceptive orchids entice and then defraud their specific (one or few) insect pollinators by emitting volatiles that mimic the sex pheromones of female insects or the presence of the pollinators’ prey, oviposition sites, shelter, and rendezvous points. These volatiles can consist of commonly occurring floral compounds in unusual blends and/or as unusual compounds that are uncommon in nature (Bohman et al., 2016). This chemical blends likely serve as sensory private channels that promote specialized plant-pollination relationships in plants, especially in the *Orchidaceae* (Raguso, 2008; Schäffler et al., 2015). The ability of plants to rapidly evolve new pathways or to fine-tune existing pathways for synthesizing these unique floral volatiles blends may hold a key to the widespread evolution of deceptive pollination (Schlüter and Schiestl, 2008; Schiestl and Schlüter, 2009). The mechanisms underpinning this evolution likely include gene duplication and divergence, convergent (and repeated) evolution, and alteration/loss of gene expression and enzyme activities (Gang, 2005).

In this perspective, we first illustrate some examples of the diverse deceptive pollination strategies of the orchids and highlight their chemical diversity. Next, we explore the potential metabolic pathways and biosynthetic steps that might be involved in the production of the often-unusual compounds. Finally, we offer recommendations that may accelerate the discovery of the biochemical pathways in this challenging but intriguing study systems.

## Volatile Diversity and Pollinator Specificity in Deceptive Orchids

**FIGURE 1:**
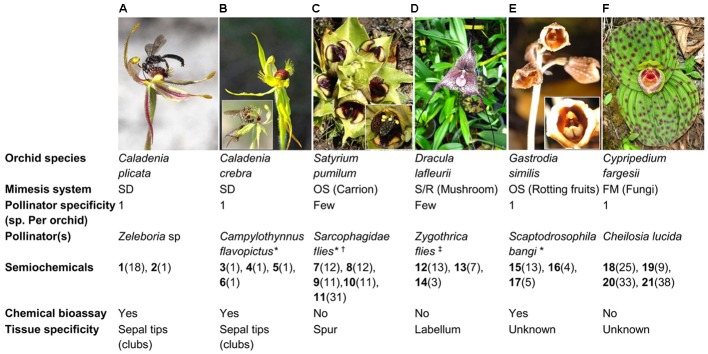
An illustration of the diversity of orchids employing different deceptive pollination strategies. **(A)** *Caladenia plicata*, **(B)** *Caladenia crebra*, **(C)** *Satyrium pumilum*, **(D)** *Dracula lafleurii*, **(E)** *Gastrodia similis*, and **(F)** *Cypripedium fargesii*. For each orchid species, information on the pollinator(s) (i.e., specificity, species) and volatiles involved (i.e., type, distribution, field bioassay confirmation, tissue specificity) are summarized. Parenthesis for each compounds listed indicate their distribution as floral volatile constituents in angiosperm families surveyed by Knudsen et al. (2006) or as identified in the recent studies discussed in this perspective. A threshold of 50% occurrence (≥45 of the 90 families) determines ‘common’ floral volatiles as per Knudsen et al. (2006). SD, Sexual deception; FM, Food microorganism; OS, Oviposition site; S/R, Shelter/rendezvous; (**1**) (*S*)-β-citronellol; (**2**) 2-hydroxy-6-methylacetophenone; (**3**) 2-(methylthio)phenol; (**4**) 2-(methylthio)benzene-1,4-diol; (**5**) 4-hydroxy-3-(methylthio)benzaldehyde; (**6**) 4-(hydroxymethyl)-2-(methylthio)phenol; (**7**) dimethyl disulfide; (**8**) dimethyl trisulfide; (**9**) 2-heptanone; (**10**) *p*-cresol; (**11**) indole; (**12**) 1-octen-3-ol; (**13**) 3-octanone; (**14**) 3-octanol; (**15**) ethyl acetate; (**16**) ethyl 2-methylpropanoate; (**17**) methyl 2-methylpropanoate; (**18**) 3-methyl-1-butanol; (**19**) 2-ethyl-1-hexanol; (**20**) 1-hexanol; (**21**) benzyl acetate; ^∗^ see inset; ^†^ (e.g., *Sarcophaga redux*, *Sarcophaga guillarmodi*, and many other indeterminate spp.); ^‡^ (e.g., *Zygothrica antedispar* and many other indeterminate species of *Zygothrica vittatifrons* group). All images have been reproduced with permission from the respective copyright holders. Please refer to the Section “Acknowledgments” for image credits.

Two key features characterize many of the deceptive orchid mimicry examples: (1) Floral volatiles play a pivotal role in the interaction. (2) Pollinator specificity, whereby only one or a few pollinator species are involved, is frequent. Furthermore, while morphology and pigmentation may also play important roles (Schlüter and Schiestl, 2008; Schiestl and Schlüter, 2009), this pollinator specificity is often strongly controlled by chemistry. Below we explore these two themes for some exemplars of deceptive pollination (**Figure 1**).

Although representing just a fraction of the many cases of deceptive pollination, sexual deception is one of the best chemically known pollination system in orchids. In this highly specific system (often just one pollinator), an extraordinary diversity of plant chemicals are now confirmed as the female sex pheromone mimics. These include alkenes and alkanes, uncommon keto and hydroxycarboxylic acids, cyclohexan-1,3-diones, pyrazines, and (methylthio)phenols as active semiochemicals in various sexually deceptive orchids (Bohman et al., 2016, 2017a,b).

Extreme pollinator specificity has also been reported for orchids that mimic oviposition sites (Martos et al., 2015) and food microbe sources (Ren et al., 2011) via emitting floral volatiles that are associated with rotting fruits and food microorganisms, respectively. Even in cases where pollinator specificity is less extreme, orchids that mimic oviposition, shelter, and rendezvous sites often attract just a specific subset of the many potential pollinators by using floral volatiles associated with carrion or fungi (van der Niet et al., 2011; Policha et al., 2016).

## Case Studies of Candidate Floral Volatiles for Deceptive Pollination and Floral Tissue Specificity

The monoterpene alcohol, β-citronellol, in a unique blend with 2-hydroxy-6-methylacetophenone, a volatile only known from *Caladenia plicata* flowers (**Figure 1A**), play a crucial role in deceptively attracting the mate-seeking male wasp pollinators of just one species (Xu et al., 2017). Production of these compounds is restricted to the sepal tips (clubs) of the flower. In *Caladenia crebra*, flowers emit (methylthio)phenols such as 2-(methylthio)phenol, 2-(methylthio)benzene-1,4-diol, 4-hydroxy-3-(methylthio)benzaldehyde, and 4-(hydroxymethyl)-2-(methylthio)phenol (**Figure 1B**) to sexually deceive its single pollinator, male *Campylothynnus flavopictus* wasp (Bohman et al., 2017a). Productions of these (methylthio)phenols are also restricted to the sepal tips.

Flowers of the fly-pollinated *Satyrium pumilum* orchids emit a cocktail of six compounds (**Figure 1C**) containing sulfurous oligosulfides such as dimethyl disulfide (DMDS) and dimethyl trisulfide (DMTS). Emission of these volatiles is also tissue-specific, in this case to the flower spur. Both DMTS and/or DMDS are predicted to be the key olfactory cue for attracting the flesh-eating fly pollinators of *Satyrium pumilum* flowers (van der Niet et al., 2011), consistent with bioassay evidence in other plants (Stensmyr et al., 2002; Shuttleworth and Johnson, 2010; Zito et al., 2014). In the *Dracula* orchid, *Dracula lafleurii*, the labellum acts as both a visual and an olfactory mimic of mushrooms that often grow alongside these orchids (**Figure 1D**) (Policha et al., 2016). Interestingly, the labellum emits an unusual floral volatile blend of mushroom alcohols, especially (*R*)-1-octen-3-ol, which is also emitted by fruiting bodies of co-occurring fungi/mushrooms (Policha et al., 2016). Further experiments revealed that the mushroom-scented labellum is the key lure of the various drosophilid fly species. The involvement of this olfactory signal may be relevant to other *Dracula* orchid species such as *D. chestertonii*, *D. vampira*, *D. chimaera* (Kaiser, 2006).

The above examples highlight floral volatiles of known tissue specificity, and it is likely that tissue specific volatile production will characterize deceptive pollination systems generally. Indeed, tissue-specific emission of floral volatiles is a hallmark of many plants, including rewarding species (Muhlemann et al., 2014). Although the precise location of volatile production is unknown, *Gastrodia similis* orchids illustrate an interesting case of rotting fruit mimicry. Flowers emit a scent reminiscent of several host fruits of its sole pollinator, the drosophilid fly *Scaptodrosophila bangi* (**Figure 1E**). The active semiochemicals consist of a blend of three fatty-acid esters, ethyl acetate, ethyl isobutyrate, and methyl isobutyrate (Martos et al., 2015). Meanwhile, *Cypripedium fargesii* has been hypothesized to mimic fungi. Whole flowers emit volatiles normally associated with black mold fungus-infected plant tissue, especially 3-methylbutanol (Ren et al., 2011), and are postulated to be the key cues used to deceptively attract fungi-feeding hoverflies as pollinators. This hypothesis remains to be confirmed by bioassays (**Figure 1F**).

## Biosynthesis of Floral Volatiles: Challenges and Lessons for Moving Forward

There has been great progress in deciphering the biochemical and genetic processes underlying the synthesis of floral volatile classes present throughout angiosperms, such as the terpenoids, phenylpropanoids/benzenoids, and volatile fatty acid derivatives (Dudareva et al., 2013). Much, however, remains to be determined, which is not surprising since the number of recognized floral volatiles now stands at ca. 1700 compounds (Knudsen et al., 2006). Furthermore, many other volatiles not yet reported in flowers are known to be synthesized elsewhere in the plant, such as in leaves and roots (Pichersky and Gershenzon, 2002; Dudareva et al., 2013), and it is likely that many of these will eventually be detected in flowers as well (Schiestl, 2010).

Unlocking the biosynthesis of floral volatiles involved in deceptive pollination systems is particularly challenging. Many of the deceptive floral scent compounds in these plants have a limited taxonomic distribution. Genetic resources (e.g., genome and transcriptome sequence databases) that can be invaluable in forward and reverse genetic approaches to elucidate biochemical pathways are rarely available. Often, these plants cannot even be grown in cultivation for a full life-cycle, and biological material has to be collected in nature during the short period of time in the year when the plants are in bloom. Nonetheless, for one recently discovered semiochemical involved in sexual deception, (*S*)-β-citronellol, the complete biosynthetic pathway has now been elucidated in the sexually deceptive orchid *C. plicata* (Xu et al., 2017).

Despite much interest in β-citronellol, given its sporadic but diverse taxonomic distribution in plants, its biosynthesis remained unknown until the work done in this non-model organism. Earlier work in several model plants such as tomato (Davidovich-Rikanati et al., 2007) and ginger (Iijima et al., 2014) did establish that geraniol was the key precursor of (*S*)-β-citronellol, but subsequent steps were not determined. To identify the genes and enzymes involved in the conversion of geraniol to β-citronellol in *C. plicata*, *de novo* transcriptome assembly and differential expression analysis between club (active) and column (non-active) tissue transcriptomes were carried out. A candidate geraniol synthase gene was quickly identified based both on differential expression and membership in the terpene synthase (TPS) gene family, and the protein encoded by this gene was biochemically demonstrated to catalyze the formation of geraniol from geranyl diphosphate (Xu et al., 2017). The analysis also identified one highly expressed alcohol dehydrogenase (*CpADH3*) and one tissue-specific double-bond reductase (*CpGER1*) transcript as promising candidates. These transcripts belonged to the gene/protein family of interests (i.e., dehydrogenase and reductase) and/or possessed the desired profile of strong differential expression. Contrary to previous predictions of a one-step conversion of geraniol to β-citronellol (Hsiao et al., 2006; Schwab and Wust, 2015), subsequent biochemical assays for *Cp*ADH3 and *Cp*GER1 revealed that β-citronellol biosynthesis from geraniol proceeds in three steps, beginning with the oxidation of geraniol to geranial by *Cp*ADH3, enantioselective reduction of geranial to (*S*)-β-citronellal by *Cp*GER1, and a further reduction of (*S*)-β-citronellal to (*S*)-β-citronellol by *Cp*ADH3 (Xu et al., 2017).

The breakthrough in the elucidation of the biosynthesis of β-citronellol in this non-model plant species was aided by several key factors: (1) Considerable relevant background research in other plants. (2) It was well-established that geraniol was a precursor of β-citronellol. (3) β-Citronellol along with the second active compound, 2-hydroxy-6-methylacetophenone were the dominant floral volatiles. (4) Production of the compound was tissue specific. (5) Thus, strategically targeted contrasting active and non-active tissue transcriptomes were produced, allowing the downstream differential expression analysis, identification of the candidate genes involved, and confirmation of gene function. This combination of just a few dominant components of floral scent (in an often simple floral bouquet) and tissue specific production are common features of the deceptive pollination examples illustrated earlier (**Figure 1**). Thus, differential expression of active and non-active tissue transcriptomes has the potential to rapidly aid identification of candidate genes.

## Inisights Into the Biosynthesis of Semiochemicals Involved in Deceptive Pollination Systems

**FIGURE 2:**
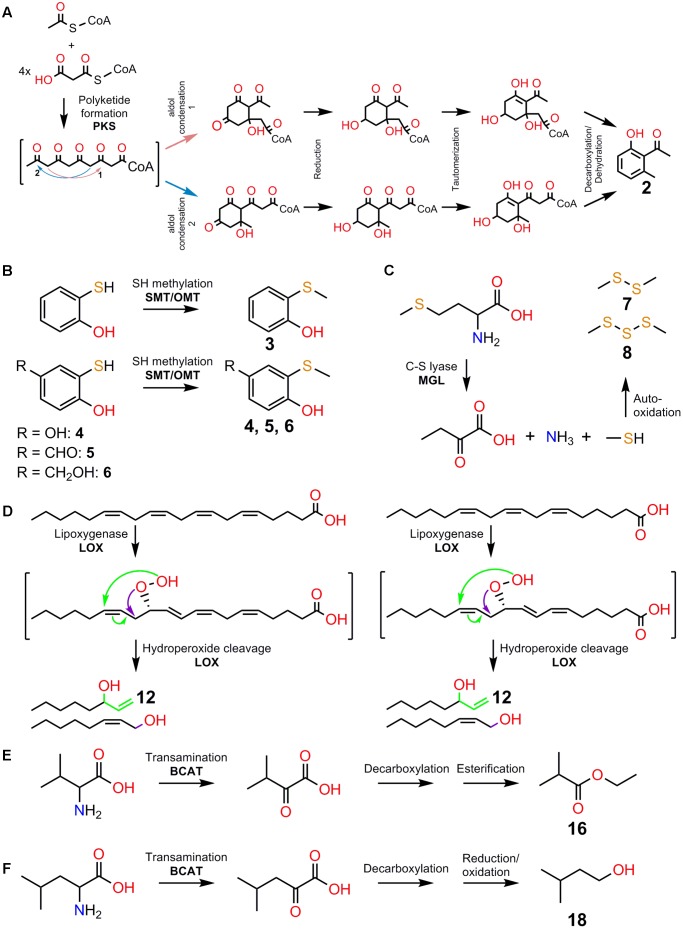
A subset of volatile attractants involved in deceptive orchid pollination and their potential biosynthetic pathways. **(A)** Formation of 2-hydroxy-6-methylacetophenone (**2**) using one acetyl-CoA and four malonyl-CoA starter units via a polyketide synthase pathway involving aldol condensation, reduction, tautomerization, and decarboxylation/dehydration reactions. Involvement of polyketide synthase (PKS) is indicated. **(B)** Formation of the (Methylthio)phenols, 2-(methylthio)phenol (**3**), 2-(methylthio)benzene-1,4-diol (**4**), 4-hydroxy-3-(methylthio)benzaldehyde (**5**), and 4-(hydroxymethyl)-2-(methylthio)phenol (**6**) via sulfhydryl/thiol (-SH) methylation of sulfhydryl-containing precursors. Involvement of *S*-methyltransferase (SMT) and *O*-methyltransferase (OMT) is indicated. **(C)** Formation of dimethyl disulfide (**7**) and dimethyl trisulfide (**8**) via auto-oxidation of methanethiol. The action of C-S lyase activity on methionine produces α-ketobutyrate, ammonia, and methanethiol. Involvement of methionine gamma-lyase (MGL) is indicated. **(D)** Formation of 1-octen-3-ol (**12**) via the lipoxygenase (LOX) pathway with arachidonic acid and γ-linolenic acid as precursors. Involvement of LOX enzymes is indicated. Note that LOX activities on arachidonic acid and γ-linolenic acid precursors can give rise to 12-Hydroperoxyeicosatetraenoic acid (12-HPETE) and 10-γ-hydroperoxyoctadecatrienoic acid (10-γ-HPOTE) products, respectively. 12-HPETE and 10-γ-HPOTE serve as intermediates for the formation of compounds such as **12** and (2*Z*)-octen-1-ol via unusual LOX fatty acid chain-cleaving lyase activities. **(E)** Formation of ethyl 2-methylpropanoate (**16**) via decarboxylation and esterification of α-ketoisovalerate intermediate. **(F)** Formation of 3-methyl butanol (**18**) via decarboxylation and reduction/oxidation of α-ketoisocaproate intermediate. Transamination/deamination of L-valine and L-leucine leads to the formation of α-ketoisovalerate and α-ketoisocaproate, respectively. Involvement of branched-chain aminotransferase (BCAT) enzymes is indicated. Note that α-ketoisovalerate can serve as alternative intermediate for (**18**). The numbering of compounds are kept consistent as listed in **Figure 1** for clarity.

Motivated by the success of the elucidation of (*S*)-β-citronellol biosynthesis in a non-model system, here we explore the biosynthetic pathways involved in 2-hydroxy-6-methylacetophenone, (methylthio)phenols, dimethyl di- and tri-sulfide, 1-octen-3-ol, ethyl 2-methylpropanoate, and 3-methyl butanol formation (**Figures 2A–F**) by drawing on the literature to establish some plausible hypotheses for the biosynthesis of some compounds involved in deceptive pollination.

### Biosynthesis of 2-Hydroxy-6-Methylacetophenone

Compared to β-citronellol, 2-hydroxy-6-methylacetophenone is a rare floral volatile presently only known in the flowers of five *Caladenia* orchids – *Caladenia attingens*, *C. ferruginea, C. pectinata, C. thinicola*, and *C. plicata* (Xu et al., 2017). Two alternative biosynthesis hypotheses have been proposed – a polyketide biosynthetic route based on evidence from radiolabeling studies in an ant (Tecle et al., 1986) or via coenzyme A-dependent β-oxidation of phenylpropanoid precursors (Negrel and Javelle, 2010). Here, we present a hypothesized pathway for the formation of 2-hydroxy-6-methylacetophenone via a polyketide biosynthetic route (**Figure 2A**). The polyketide biosynthetic route deserves attention as a strong tissue-specific differential expression of one polyketide synthase (*CpPKS1*) is present (Xu et al., 2017).

### Biosynthesis of (Methylthio)phenol

Plant enzymes catalyzing the methylation of sulfhydryl/thiol (-SH) group have been previously reported, for example, *S*-methyltransferase 1 (CrSMT1) in *Catharanthus roseus* (Coiner et al., 2006). This enzyme is capable of methylating different sulfhydryl-containing aliphatic and aromatic compounds with different efficiencies, and is closely related to plant *O*-methyltransferases (**Figure 2B**). Given that *O*-methyltransferase are widely distributed in plants as large gene families (Gang, 2005) and that only a few substitutions in key residues are sufficient for converting an *O*-methyltransferase into an *S*-methyltransferase (Coiner et al., 2006), close attention should be given to *O*-methyltransferase gene families in *Caladenia crebra*. Under this hypothesis, the biosynthesis of 2-(methylthio)phenol would be achieved by the methylation of 2-hydroxythiophenol (**Figure 2B**). Potential *O*-/*S*-methyltransferase homologs may also evolve unique capacities to accept the different sulfhydryl-containing precursors with different side chains (e.g., –CHO, –CH_2_OH, –OH) and/or at various positions, which may otherwise interfere with substrate acceptability as demonstrated for CrSMT1 (Coiner et al., 2006).

### Biosynthesis of Dimethyl Disulfide and Dimethyl Trisulfide

Biochemical and genetic evidence for the formation of DMDS and DMTS has been established in several plant species (Boerjan et al., 1994; Tulio et al., 2002; Rébeillé et al., 2006; Gonda et al., 2013). First, formation of methanethiol, a highly unstable and reactive compound, requires the sulfur-containing amino acid methionine as precursor. Next, methanethiol autooxidises to DMDS and DMTS (**Figure 2C**). Thus, the enzyme methionine gamma-lyase (MGL) which catalyzes the catabolism of methionine to form α-ketobutyrate, methanethiol, and ammonia (Gonda et al., 2013) is a strong candidate for DMDS and DMTS biosynthesis in *Satyrium pumilum* flowers. Alternatively, methanethiol could also be formed via methylation of bisulfide ion ([SH]-) as demonstrated by several *Brassicaceae* thiol methyltransferase (Attieh et al., 2002; Itoh et al., 2009; Nagatoshi and Nakamura, 2009). However, this pathway seems unlikely in orchids which are non glucosinolate-producing plants (Iranshahi, 2012).

### Biosynthesis of 1-Octen-3-ol

Plant lipoxygenases (LOXs) belong to large gene families and commonly catalyze the stereo-specific oxygenation of octadecanoid precursors at positions C9 (9-LOX) and C13 (13-LOX) resulting in the formation of various 9- and 13-hydroperoxy intermediates, respectively (Feussner and Wasternack, 2002). Biochemical studies have shown that the moss *Physcomitrella patens* possesses a multi-functional LOX enzyme (*PpLOX1*) with unique fatty acid chain-cleaving lyase activities capable of producing 1-octen-3-ol via 12- and 10-hydroperoxy intermediates (**Figure 2D**), the products of arachidonic acid and γ-linolenic acid precursors, respectively (Senger et al., 2005). 1-Octen-3-ol has also been reported to be specifically induced in reproductive (Savoi et al., 2017) and vegetative (Wichard et al., 2004) tissues of plants during abiotic stress. A tissue- and/or stress-specific expression of a unique *LOX*, may be hypothesized for 1-octen-3-ol biosynthesis in the labella of *Dracula lafleurii*.

### Biosynthesis of Ethyl 2-Methylpropanoate and 3-Methyl Butanol

Catabolism of branched chain amino acids (BCAA) such as L-isoleucine, L-leucine, and L-valine, is key to the formation of many BCAA-derived volatiles including ethyl 2-methylpropanoate and 3-methylbutanol (Gonda et al., 2010, 2013; Kochevenko et al., 2012), with respective α-keto acids serving as key intermediates (**Figures 2E,F**). The branched-chain aminotransferase (BCAT) enzymes that catalyze the transamination/deamination of the amino acid precursor to α-keto acids have also been characterized in several fruit crops rich in BCAA-derived volatiles (Gonda et al., 2010; Maloney et al., 2010). Strong evidence implicating BCATs in 3-methylbutanol formation (**Figure 2F**) has also been obtained in transgenic over-expression studies of tomatoes (Kochevenko et al., 2012). Although the tissue-specific distribution of ethyl 2-methylpropanoate and 3-methylbutanol in the flowers of *Gastrodia similis* and *Cypripedium fargesii*, respectively remains to be determined, specific BCATs isoforms may be relevant for their biosynthesis (**Figures 2E,F**).

## Future Directions and Final Remarks

High throughput sequencing methodology is an emerging tool for profiling gene expression at a genome-wide scale in non-model plants. The adoption of this technique, especially the sequencing of mRNA from floral tissues and *de novo* transcriptome reconstruction, to prioritize candidate genes and pathways involved in the biosynthesis of deceptive semiochemicals in several orchids have already been carried out (Sedeek et al., 2013; Wong et al., 2017; Xu et al., 2017). To initiate the prioritization of candidate genes and pathways, targeted and strategic transcriptome analysis of active (scent-producing) and non-active flower organs and tissues will often be the first key step. Differential expression analysis between the active and non-active tissues can then be performed, where the latter provides an excellent baseline for identifying differentially expressed genes in the active tissues. This approach was used to the authors’ advantage in the breakthrough on (*S*)-β-citronellol biosynthesis (Xu et al., 2017), and may hold potential for elucidating other biosynthetic pathways. When closely related orchid species also employs a common volatile, such as 1-octen-3-ol in some *Dracula* orchids (Kaiser, 2006), transcriptome analysis across species with the goal of identifying shared gene expression and metabolic pathways, may also prove informative.

To provide additional support to the candidates prioritized from targeted/strategic transcriptome analysis highlighted above, an integrated network analysis can be performed. When simultaneous profiling of deceptive volatiles across diverse conditions and their corresponding sample transcriptomes is feasible, such metabolic profiles can be used as ‘guides’ or ‘baits’ to infer functionally associated genes that satisfy a given similarity threshold (e.g., correlation and mutual information). This approach is based on the well-established observations that genes and metabolites involved in related processes often have parallel expression/accumulation dynamics across a range of conditions such as tissues and developmental stages (Schilmiller et al., 2012; Wong and Matus, 2017).

A complementary strategy should also include molecular evolutionary analysis. For example, testing for gene duplication and selection signatures on hypothesized pathway genes in a phylogenetic context is often useful. Such an analysis provided critical clues toward the identification of the candidate stearoyl-acyl carrier protein desaturase enzymes involved in 7-, 9-, and 12- alkene biosynthesis in sexually deceptive *Ophrys* orchids (Schlüter et al., 2011; Sedeek et al., 2016).

## Conclusion

As critical first step, here we have drawn on prior biochemical knowledge from other systems to build plausible hypotheses on the biosynthesis of some volatiles involved in deceptive pollination. We have also highlighted the promising approaches that will allow these hypotheses to be tested. While orchids represent particularly challenging systems, as neither their biochemistry nor genomes and transcriptomes have been extensively characterized, these approaches are making the biochemical investigation of deceptive chemicals in orchids both feasible and rewarding. Beyond deceptive orchids, these approaches serve as valuable guidelines for other plants, including rewarding species, particularly those species employing unique floral volatile blends for pollinator attraction.

## Author Contributions

DW conceived the article, planned its structure, discussed the literature, and wrote the article with assistance from EP and RP. All authors have read and approved the paper.

## Conflict of Interest Statement

The authors declare that the research was conducted in the absence of any commercial or financial relationships that could be construed as a potential conflict of interest.
